# Inhibitory Effects of Fermented Sprouted Oat Extracts on Oxidative Stress and Melanin Overproduction

**DOI:** 10.3390/antiox13050544

**Published:** 2024-04-29

**Authors:** Hyeijin Cho, Jisun Yang, Ji Young Kang, Kyung Eun Kim

**Affiliations:** 1 Department of Health Industry, Sookmyung Women’s University, Seoul 04310, Republic of Korea; chj@sookmyung.ac.kr (H.C.); ellykang@sookmyung.ac.kr (J.Y.K.); 2 Department of Cosmetic Sciences, Sookmyung Women’s University, Seoul 04310, Republic of Korea; jas11070@naver.com

**Keywords:** *Avena sativa* L., sprouted oat, postbiotics, antioxidant, anti-melanogenesis, anti-hyperpigmentation

## Abstract

Hyperpigmentation occurs due to irregular secretion of melanin pigment in the skin. This can affect quality of life depending on its severity, so prevention and management are essential. Oats (*Avena sativa* L.), a grain consumed worldwide, are known to offer improved health benefits upon germination and fermentation. This study is aimed to investigate the protective effects of lactobacilli-fermented sprouted oat extracts on oxidative stress and melanin overproduction in vitro. The anti-melanogenic effect was investigated using melanin content and tyrosinase activity assays in B16F10 cells, as well as a mushroom tyrosinase-based enzyme inhibition assay. The results showed that *L. casei*-fermented oat extracts were the most effective for reducing melanin formation by reducing the mRNA expression of microphthalmia-associated transcription factor, tyrosinase, and tyrosinase-related protein 2. Furthermore, *L. casei* fermentation was effective in improving the total phenolic, flavonoid, and avenanthramide A contents of sprouted oat extracts. The results also demonstrated the antioxidant effects of *L. casei*-fermented sprouted oat extracts in promoting DPPH radical-scavenging activity, superoxide dismutase-like activity, and reduction in reactive oxygen species levels. Overall, the findings indicate that fermented sprouted oat extracts are promising candidates for antioxidant and anti-hyperpigmentation treatments.

## 1. Introduction

The skin offers an immunological microenvironment that protects the body by forming a barrier against the external environment [1,2]. Human skin comprises epidermal, dermal, and hypodermal (subcutaneous fat) layers, and homeostasis is maintained through the functions of various skin cells [3]. Melanocytes, present in the basal layer of the epidermis, synthesize melanin pigments and transfer them to keratinocytes in the form of melanosomes to protect the human body from ultraviolet (UV) radiation [4]. Moderate UV exposure can induce the synthesis of vitamin D and reduce the risks of obesity and diabetes [3]. However, excessive exposure to UV light causes undesirable changes in skin pigmentation, such as hyperpigmentation and freckle formation, due to melanin overproduction [5,6,7,8,9].

Melanogenesis is a complex process in which melanin pigments are produced by melanocytes and transported by melanosomes [8,10]. The process is initiated by tyrosinase, which plays a vital role as a rate-limiting enzyme in the early steps of the biosynthesis pathway that produces melanin pigment [11]. Tyrosinase catalyzes the oxidation of tyrosine to 3,4-dihydroxyphenylalanine (DOPA) and dopaquinone, a process regulated by microphthalmia-associated transcription factor (MITF) [12,13,14]. Dopaquinone is naturally oxidized and converted into dopamine [12]. Subsequently, the reaction product dopachrome, which is formed by oxidation and enzymatic reactions, is converted to 5,6-dihydroxyindole-2-carboxylic acid (DHICA) by tyrosinase-related protein (TRP)-2, and dark-colored eumelanin is produced by TRP-1 [11,15,16,17,18]. In addition to its role in melanin synthesis, MITF is also involved in the survival and differentiation of melanocytes, and MITF upregulation has been identified in melanoma [13,19,20]. Conversely, pale skin in the elderly has been associated with reduced tyrosinase activity and melanogenesis due to aging [21,22]. Therefore, reducing the expression of MITF, tyrosinase, and tyrosinase-related enzymes can inhibit the overproduction and accumulation of melanin and prevent hyperpigmentation.

Oxidative stress is also recognized as a factor in several melanin-related conditions, such as skin hyperpigmentation [23,24]. Oxidative stress arises from an imbalance between the generation and elimination of reactive oxygen species (ROS) [24]. Increased ROS are neutralized by antioxidants generated through activation of the Nrf2-ARE pathway. Nuclear factor erythroid 2-related factor 2 (Nrf2) dissociates and translocates into the nucleus upon exposure to oxidative stress and binds to the antioxidant response element (ARE) of DNA. Transcription of antioxidant and detoxification genes through this pathway protects cells from oxidative stress and contributes to maintaining the redox balance of cells. However, cells under oxidative stress can inhibit the function of antioxidant enzymes such as superoxide dismutase (SOD) and can induce vitiligo via melanocyte apoptosis [25]. Intracellular ROS also induce degenerative changes in cells by damaging biological macromolecules, including proteins and DNA, and altering skin pigmentation with age, as seen in senile vitiligo and senile lentigo [21,26]. In addition, ROS stimulate melanocytes to produce excessive melanin pigments by promoting tyrosinase enzyme activity, leading to the formation of pigmented skin spots [27,28,29]. These examples demonstrate that oxidative stress is an important factor underlying antioxidant system damage and melanocyte activity in skin cells. Therefore, the prevention of skin aging and excessive pigmentation can be expected by applying substances with antioxidant effects that remove ROS.

Oats (*Avena sativa* L.) are consumed worldwide due to their various health benefits, including antioxidant, anti-UVB photoaging, and anti-inflammatory effects [24,30,31]. They are also consumed as sprouted grains, which can be grown without pesticides or chemical fertilizers because of the short germination period of *Avena sativa* L. [32]. During germination, grains such as oats absorb moisture, soften, and undergo enzyme activation [33,34,35]. Activated enzymes lead to changes in the release of nutrients and increase the content of health-promoting compounds [35,36,37]. Oats are rich in nutrients and contain avenanthramides, a polyphenol not found in other grains [30]. The contents and activities of avenanthramides and related enzymes in oats were altered by hydration and germination, and sprouted oats have been found to contain more phytochemicals including avenanthramides than typical dried oats [34,38,39,40,41]. Several studies have reported that avenanthramides exert anti-inflammatory and anti-cancer effects. Particularly, avenanthramide A has been demonstrated to have an anti-inflammatory effect by inhibiting the production of NO through reducing iNOS expression, an inflammatory factor [42]. Type A avenanthramide also induced cellular senescence by upregulating the expression of miR-129-3p, and avenanthramide C was shown to inhibit COX-2 expression in A549 cells and to induce cellular senescence in CRC cells by attenuating β-catenin-mediated transactivation [43,44,45]. Furthermore, sprouted oats are known to regulate oxidative stress, blood pressure, and low-density lipoprotein cholesterol in blood vessels [34,38,46]. However, the effect of sprouted oats on skin hyperpigmentation due to oxidative damage has not been investigated.

The composition and activities of health-promoting substances, such as sprouted oats, can be enhanced by processes such as fermentation. Fermentation is a processing technology that controls the microbial growth and enzyme activity of natural substances, improving their health benefits [47,48]. Fermentation can improve stability and biochemical quality by regulating the composition of various microorganisms [49,50]. For example, fermentation using *Lactiplantibacillus plantarum* provides antibacterial agents that increase the storage period of products and improve stability. Milk fermented using *Lactobacillus helveticus* NS8 inhibits melanin production in murine melanoma cell lines and prevents both the enzymatic activity of tyrosinase and the protein expression necessary for melanin synthesis [51,52,53]. In addition, probiotics developed using fermentation techniques have been reported to offer skin health benefits through pathogen destruction, immunoregulation, and enhanced migration and functionality in fibroblasts and epithelial cells [54,55]. Moreover, postbiotics contain biotechnological extracts from microorganisms and exhibit immunomodulatory, anti-inflammatory, and tolerogenic activities in atopic dermatitis models [56].

Sprouted oats are known to exhibit boosted nutritional value due to germination, and fermentation is known to improve the health benefits of many natural substances; however, the improvement of nutritional and health attributes in sprouted oats through fermentation has not been investigated [39]. Therefore, this study aimed to assess the antioxidant and anti-melanogenic properties of fermented sprouted oat extract. For this purpose, sprouted oat extracts were fermented with *Lactiplantibacillus plantarum* subsp. *plantarum*, *Lacticaseibacillus casei*, *Lacticaseibacillus rhamnosus*, and *Lactobacillus gasseri*. This study was based on the hypothesis that the lactobacilli-fermented sprouted oat extracts would be more effective in anti-hyperpigmentation and antioxidants compared to non-fermented sprouted oat extracts.

## 2. Materials and Methods

### 2.1. Preparation of Fermented Sprouted Oat Extracts

The lactic acid strains *L. plantarum* subsp. *plantarum* KCTC 3108, *L. casei* KCTC 3109, *L. rhamnosus* KCTC 5033, and *L. gasseri* KCTC 3143 were purchased from the Korean Collection for Type Cultures (Seoul, Republic of Korea). These *Lactobacillus* strains were maintained in deMan–Rogosa–Sharpe (MRS) broth for lactobacilli at 37 °C. Sprouted oat powder (Naju, Republic of Korea) was dissolved at concentrations of 5% and 10% (*w*/*w*) in tertiary distilled water and sterilized in an autoclave at 121 °C for 15 min. *Lactobacillus* suspensions (1 × 10^9^ CFU/mL) were added to the mixture at a concentration of 5% (*w*/*v*) and incubated at 37 °C for 48 h in a shaking incubator at 75 rpm. The extracts were centrifuged at 9820× *g* for 20 min, filtered with a Whatman Grade No. 2 filter paper and a syringe filter with a pore size of 0.2 µm, and stored at −20 °C for subsequent experiments. Non-fermented sprouted oat extracts and tertiary distilled water were filtered under the same conditions and used as controls. All experiments were performed in triplicate to verify the results.

### 2.2. Cell Culture

B16F10 murine melanoma cell lines were purchased from the American Type Culture Collection (Manassas, VA, USA). B16F10 cells were cultured in complete Dulbecco’s modified Eagle’s medium (DMEM; Cytiva Life Sciences, Logan, MA, USA) supplemented with 10% fetal bovine serum and 1% penicillin–streptomycin and incubated at 37 °C with 5% CO_2_ in a humidified incubator.

### 2.3. Cell Viability Assay

A Cell Counting Kit 8 (CCK-8) assay was performed to measure cell viability. CCK-8 solution was purchased from Dojinbio (Seoul, Republic of Korea). B16F10 cells were grown in 96-well plates and incubated at 37 °C with 5% CO_2_ for 24 h. Non-fermented and lactobacilli-fermented sprouted oat extracts were added at ratios of 1:200, 1:100, and 1:50 of total volume. Cell viability was determined after 24, 48, and 72 h incubation at 37 °C with 5% CO_2_. Two hours before measurement, 20 µL of CCK-8 solution was added to the cells and reacted in an incubator. Samples were then shaken for 10 s, and the absorbance was measured at 450 nm using a Multiskan SkyHigh Microplate Spectrophotometer (Microplate reader; Thermo Fisher, Waltham, MA, USA).

### 2.4. Melanin Content Assay

B16F10 cells were incubated overnight at 37 °C with 5% CO_2_. Cells were treated with sprouted oat extracts at a 1:100 ratio of total volume or with 500 µg/mL arbutin (Sigma-Aldrich, St. Louis, MO, USA) as a positive control and incubated for 48 h in the absence or presence of 0.2 µM α-melanocyte-stimulating hormone (α-MSH; Sigma-Aldrich), using phenol red-free DMEM. Then, the cells were centrifuged at 15,814× *g* for 10 min after washing with phosphate-buffered saline (PBS) and lysed with 650 µL of 1 M Sodium Hydroxide (Sigma-Aldrich) with 10% DMSO (Sigma-Aldrich) at 80 °C for 1 h. To determine the relative melanin content, 200 µL medium and supernatant aliquots were transferred to 96-well plates, respectively. The absorbance was measured at 405 nm using a microplate reader.

### 2.5. Tyrosinase Activity Assay

B16F10 cells were incubated overnight in a cell culture incubator. Cells were treated with sprouted oat extracts at a 1:100 ratio of total volume or 500 µg/mL arbutin as a positive control and incubated for 48 h in the absence or presence of 0.2 µM α-MSH. To remove the cell debris, the cells were washed with PBS and centrifuged at 15,814× *g* for 10 min at 4 °C. The supernatant was discarded, and cells were treated with 100 µL of 1% (*v*/*v*) Tripton X-100 in phosphate buffer (PB, pH 6.6). The cells were then lysed on ice with shaking for 40 min and centrifuged at 15,814× *g* for 20 min. The 40 µL mixtures of 0.1 M sodium phosphate buffer (SPB, pH 7.0) and 40 µg of protein quantified via BCA protein assay (Thermo Fisher Scientific) were transferred to 96-well plates with 200 µL of 10 mM L-DOPA. The plate was incubated in the dark at 37 °C for 30 min, and then absorbance was measured at 475 nm using a microplate reader.

### 2.6. Mushroom Tyrosinase Inhibition Assay

Mushroom tyrosinase inhibition was measured using L-tyrosine as the substrate. The composition of the reaction mixture was 660 µL of 0.1 M SPB (pH 6.8), 60 µL of the sprouted oat extracts or 500 µg/mL arbutin, 90 µL of 7500 U/mL tyrosinase dissolved in PB (pH 6.6), and 90 µL of 15 mM L-tyrosine dissolved in 1 M HCl (Sigma-Aldrich). The reaction mixtures were incubated at 37 °C for 12 min, chilled on ice for 1 min to stop the reaction, and transferred in 150 µL aliquots to 96-well plates to measure absorbance at 490 nm using a microplate reader.

### 2.7. Reverse Transcription Quantitative Real-Time PCR (RT-qPCR)

RT-qPCR was used to determine the mRNA levels of MITF, TYR, and TRP-2 in B16F10 cells. B16F10 cells were treated with sprouted oat extracts prepared at a 1:100 ratio of total volume or with 500 µg/mL arbutin in the absence or presence of 0.2 µM α-MSH. After total cellular RNA was isolated and purified using TRIzol (Invitrogen, Waltham, MA, USA) as an agent, cDNA was synthesized using a SuperScript VILO cDNA Synthesis Kit (Invitrogen) according to manufacturer instructions. RT-qPCR was prepared using SYBR^®^ Premix Ex Taq™ (PCR Biosystems, London, UK) and was performed according to the following PCR cycling conditions using a LightCycler^®^ 96 instrument (Roche Life Science, Basel, Switzerland): Pre-incubation at 95 °C for 2 min followed by 40 cycles of denaturation at 95 °C for 5 s with annealing at 60 °C for 30 s, and extension at 72 °C for 5 min. β-actin was used as a reference to determine the levels of mRNA in the cells. The mRNA expression relative to that of β-actin mRNA was determined using the 2^−ΔΔCt^ method.

### 2.8. Phytochemical Analysis

To evaluate the total phenolic content in non-fermented and fermented sprouted oat extracts, a Folin–Ciocalteu assay was performed. The phenolic reagent (Sigma-Aldrich) was dissolved in water at 10%, and then mixed with an equal amount of 10 µL samples or gallic acid (Sigma-Aldrich) and prepared in 96-well plates. After reacting for 3 min, 150 µL of 20% Sodium Carbonate (Sigma-Aldrich) was added and the samples were left in the dark at room temperature for 1 h. The absorbance of the reaction mixture was measured at 765 nm using a microplate reader after shaking for 10 s.

The total flavonoid content of the sprouted oat extracts was determined using a colorimetric assay. At room temperature, 400 µL of sprouted oat extracts or quercetin (Sigma-Aldrich) were mixed with 30 µL of Sodium Nitrite (Sigma-Aldrich) in a 1.5 mL microcentrifuge tube and left to react for 5 min. Then, 30 µL of 10% Aluminum Chloride (Sigma-Aldrich) was added and left to react for 6 min. Next, 200 µL of 1M Sodium Hydroxide and 340 µL of tertiary distilled water were added, 200 µL aliquots were transferred to 96-well plates, and the absorbance was measured at 510 nm using a microplate reader after shaking for 10 s.

### 2.9. LC-MS/MS Analysis

The avenanthramide content in non-fermented and fermented sprouted oat extracts was detected on EVOQ Qube LC-TQ system (Bruker, Billerica, MA, USA). Avenanthramide A was separated with ACME C18 column (50 × 2.1 mm, 1.9 µm), and quantified using a standard curve of avenanthramide A. The gradient elution was performed under the conditions in Table 1.

### 2.10. DPPH Antioxidant Assay

DPPH radical-scavenging activity was measured using an OxiTec™ DPPH Antioxidant Assay Kit (Biomax, Guri, Republic of Korea). In a 1.5 mL microtube, 100 µL of sprouted oat extract or 50 µg/mL ascorbic acid (Sigma-Aldrich) was mixed with 400 µL of assay buffer and 500 µL of DPPH working solution. The reaction mixtures were left in the dark at room temperature of approximately 18 °C for 20 min, centrifuged at 15,814× *g* for 10 min, and transferred in 200 µL aliquots to 96-well plates to measure absorbance at 517 nm.

### 2.11. SOD-Like Activity Assay

SOD-like activity was evaluated using pyrogallol, which autoxidation catalyzed by superoxide radicals. The composition of the reaction mixture was 900 µL of 0.05 M Tris-HCl (pH 8.5) buffer containing 10 mM ethylenediaminetetraacetic acid (EDTA; Sigma-Aldrich), 30 µL of 7.2 mM pyrogallol (Sigma-Aldrich), and 60 µL of sprouted oat extract or 50 µg/mL ascorbic acid as a positive control. After 10 min, 30 µL of 1 M HCl was added to stop the reactions, and 200 µL aliquots of the reaction mixtures were transferred to 96-well plates to measure absorbance at 420 nm.

### 2.12. ROS Detection Assay

A 2′,7′-dichlorofluorescein diacetate (DCF-DA) assay was performed to detect ROS in B16F10 cells. After overnight incubation at 37 °C with 5% CO_2_, the cells in black 96-well plates were treated with sprouted oat extracts at a 1:100 ratio of total volume or 200 µg/mL ascorbic acid. The same amount of DMEM was used for the sample and control groups. The medium was aspirated after 24 h treatment, and then cells were rinsed with PBS. The medium was then replaced with phenol red-free DMEM containing 10 µm DCF-DA and incubated for 30 min in the dark. Then, the cells were washed three times with PBS and resuspended in 150 µL of PBS to detect fluorescence intensity at an excitation/emission wavelength of 485/535 nm using a SpectraMax i3x (Molecular Devices, San Jose, CA, USA).

### 2.13. Statistical Analysis

All experiments were performed in triplicate to verify the results. All data were analyzed using analysis of variance (ANOVA). *p* values < 0.05 were considered statistically significant. All statistical analyses were performed using GraphPad Prism version 8.0 (GraphPad Software, La Jolla, CA, USA). All results are expressed as mean ± standard deviation (SD).

## 3. Results

### 3.1. Cell Viability with Lactobacilli-Fermented Sprouted Oat Extracts

**Figure 1:**
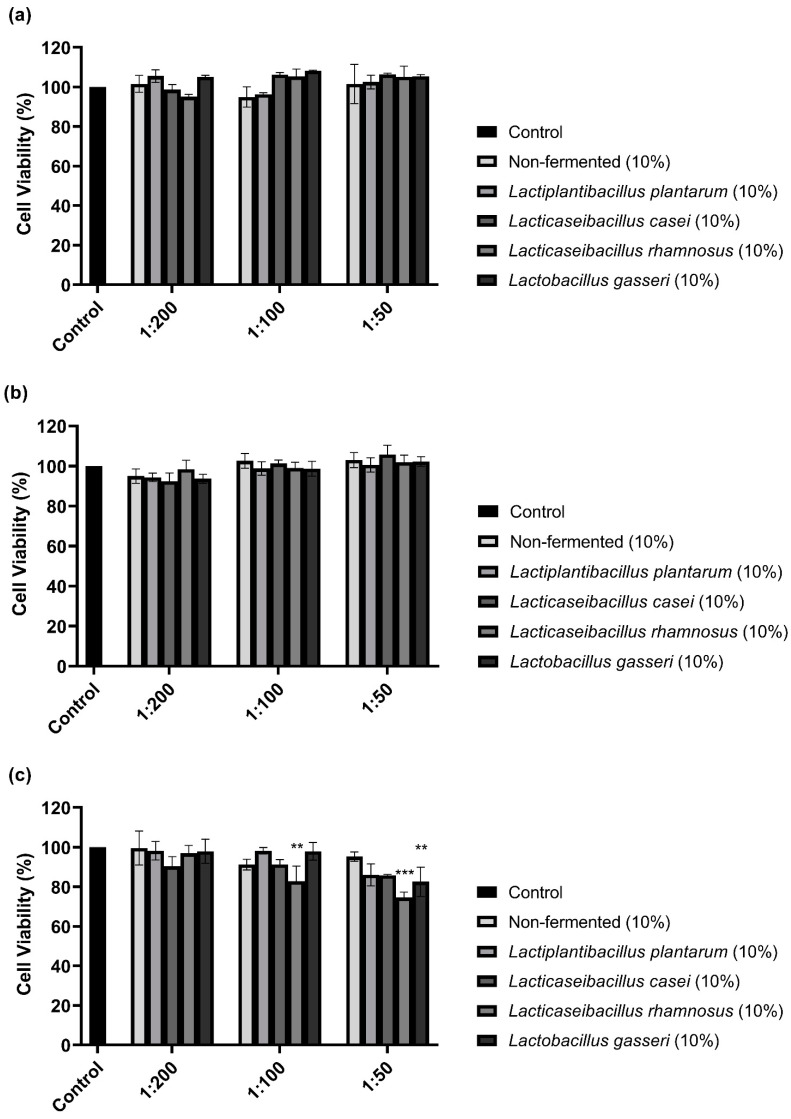
Effect of non-fermented and lactobacilli-fermented sprouted oat extracts on cell viability in B16F10 cells. B16F10 cells were treated with sprouted oat extracts at concentrations of 1:200, 1:100, and 1:50 and analyzed for (**a**) 24 h, (**b**) 48 h, and (**c**) 72 h. Error bars show means ± SD. ** *p* < 0.01, *** *p* < 0.001 compared to the untreated control group for the same treatment duration.

The CCK-8 assay performed to examine the cytotoxicity of 10% sprouted oat extracts against B16F10 cells showed that treatments with non-fermented and *Lactobacillus*-fermented sprouted oat extracts had no cytotoxic effects on B16F10 cells for 48 h (Figure 1). However, sprouted oat extracts reduced cell viability by approximately 75% at a 1:50 concentration after 72 h of treatment. Thus, further experiments on anti-melanogenic effects were performed within 48 h at an extract concentration of 1:100.

### 3.2. Melanin Synthesis and Tyrosinase Activity Inhibition by Lactobacilli-Fermented Sprouted Oat Extracts

**Figure 2:**
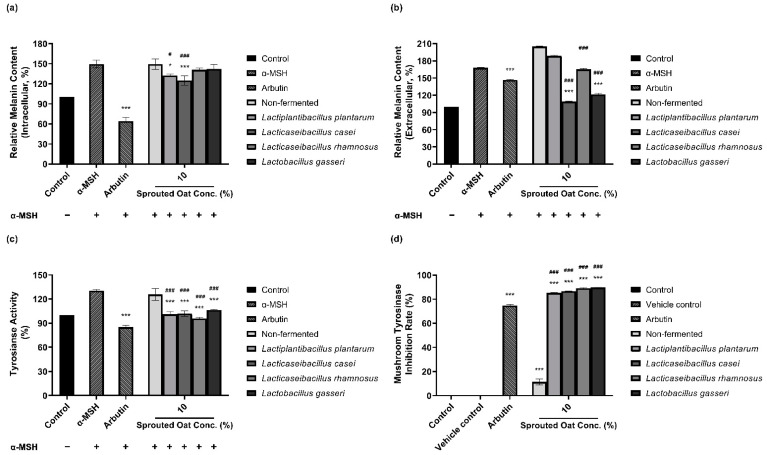
Inhibitory effects of non-fermented and lactobacilli-fermented sprouted oat extracts on melanin production in α-MSH-stimulated B16F10 cells. Relative (**a**) intracellular and (**b**) extracellular melanin contents of B16F10 cells and medium, respectively. (**c**) Intracellular tyrosinase activity. α-MSH-stimulated B16F10 cells, excluding untreated control group, were treated with non-fermented and fermented sprouted oat extracts at a concentration of 1:100 for 48 h. Arbutin (500 µg/mL) was used as a positive control. Error bars show means ± SD. * *p* < 0.05, *** *p* < 0.001 compared to the α-MSH group. # *p* < 0.05, ### *p* < 0.001 compared to the same concentration of non-fermented sprouted oat extracts. (**d**) Mushroom tyrosinase inhibition upon treatment with non-fermented and lactobacilli-fermented sprouted oat extracts. Samples were treated at a concentration of 1:15. Arbutin (500 µg/mL) was used as a positive control, and tertiary distilled water filtered under the same conditions was used as a vehicle control. Error bars show means ± SD. *** *p* < 0.001 compared to the vehicle control. ### *p* < 0.001 compared to the same concentration of non-fermented sprouted oat extracts.

The amounts of intracellular and extracellular melanin in B16F10 cells were determined to assess the inhibitory effect of fermented and non-fermented 10% sprouted oat extracts on α-MSH-induced melanogenesis. Treatment with 0.2 µM α-MSH increased the amount of intracellular melanin in B16F10 cells, as shown in Figure 2a. The amount of intracellular melanin increased to approximately 150% of the control value upon α-MSH stimulation, and non-fermented sprouted oat extracts showed no benefit in reducing the intracellular melanin content. However, extracts fermented with *L. plantarum* and *L. casei* reduced the intracellular melanin content to 132.45% and 124.98% of the control values, respectively. Additionally, Figure 2b shows the amount of extracellular melanin content increased to approximately 168% of the control value upon α-MSH stimulation and decreased to 147% with arbutin, used as a positive control. Some lactobacilli-fermented sprouted oat extracts also lowered extracellular melanin content. Notably, *L. casei*- and *L. gasseri*-fermented sprouted oat extracts reduced the extracellular melanin content to 108.93% and 121.43% of the control values, respectively.

Due to the role of tyrosinase in melanin production, the inhibitory effect of sprouted oat extracts was investigated. Sprouted oat extracts at a concentration of 10% reduced tyrosinase activity in α-MSH-induced B16F10 cells, as shown in Figure 2c. The intracellular tyrosinase activity was increased to approximately 131% of the control value with α-MSH and reduced to 85% with arbutin. Non-fermented sprouted oat extracts inhibited intracellular tyrosinase activity to 125.67% of the control value. *L. plantarum*-, *L. casei*-, *L. rhamnosus*-, and *L. gasseri*-fermented sprouted oat extracts reduced tyrosinase activity to 101.13%, 101.89%, 95.70%, and 106.30% of the control value, respectively.

Lactobacilli-fermented sprouted oat extracts also showed a higher inhibition rate than non-fermented sprouted oat extracts in a mushroom tyrosinase inhibition assay using L-tyrosine as a substrate. Figure 2d shows that the mushroom tyrosinase inhibition rate of arbutin was 74.80%, and that of non-fermented sprouted oat extracts was 11.45%. Extracts fermented with *L. plantarum*, *L. casei*, *L. rhamnosus*, and *L. gasseri* showed inhibition rates of 85.25%, 86.62%, 89.17%, and 89.73%, respectively.

Taken together, these findings reveal that *L. casei*-fermented sprouted oat extracts exhibit the most significant reduction in intracellular and extracellular melanin levels, intracellular tyrosinase activity, and mushroom tyrosinase activity (*p* < 0.001). This suggests the potential anti-melanogenic effects resulting from *L. casei* fermentation on sprouted oat extracts.

### 3.3. B16F10 Cell Viability with L. casei-Fermented Sprouted Oat Extracts

**Figure 3:**
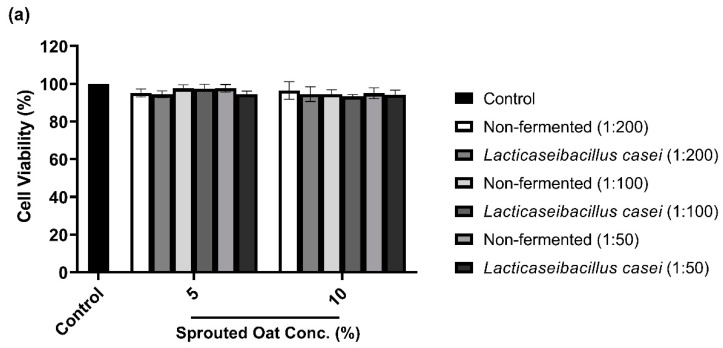

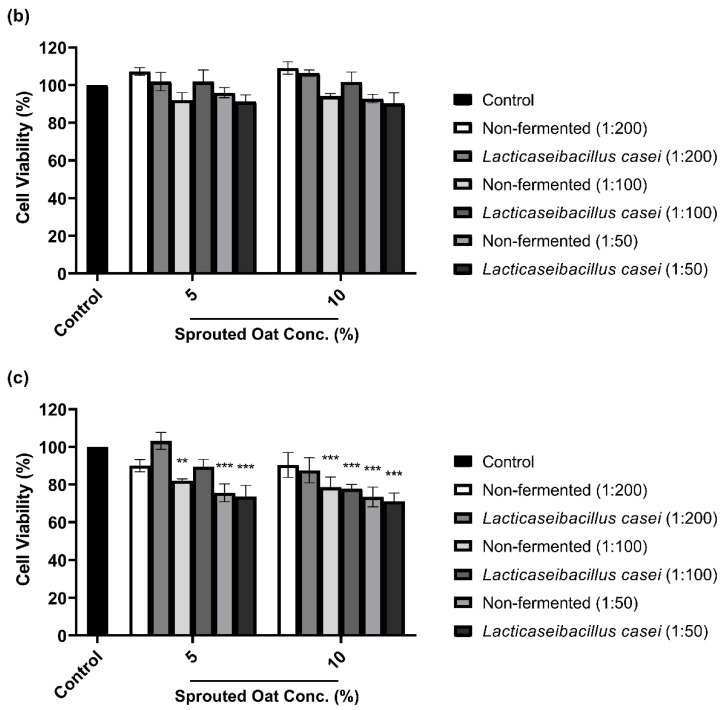
Effect of non-fermented and *L. casei*-fermented sprouted oat extracts on cell viability in B16F10 cells. Cells were treated with non-fermented and fermented sprouted oat extracts at concentrations of 1:200, 1:100, and 1:50 and analyzed for (**a**) 24 h, (**b**) 48 h, and (**c**) 72 h. Error bars show means ± SD. ** *p* < 0.01, *** *p* < 0.001 compared to the untreated control group for the same treatment duration.

To determine the maximum concentration and treatment time of *L. casei*-fermented sprouted oat extracts without cytotoxic effects on B16F10 cells, cell viability was measured using a CCK-8 assay. As shown in Figure 3a,b, B16F10 cells treated with sprouted oat extracts showed cell viability above 90% for up to 48 h. However, after 72 h of treatment, cell viability dropped to approximately 71% (Figure 3c). Therefore, further experiments on anti-melanogenesis and antioxidant effects were performed within 48 h at an extract concentration of 1:100.

### 3.4. Melanin Synthesis and Tyrosinase Activity Inhibition by L. casei-Fermented Extracts

**Figure 4:**
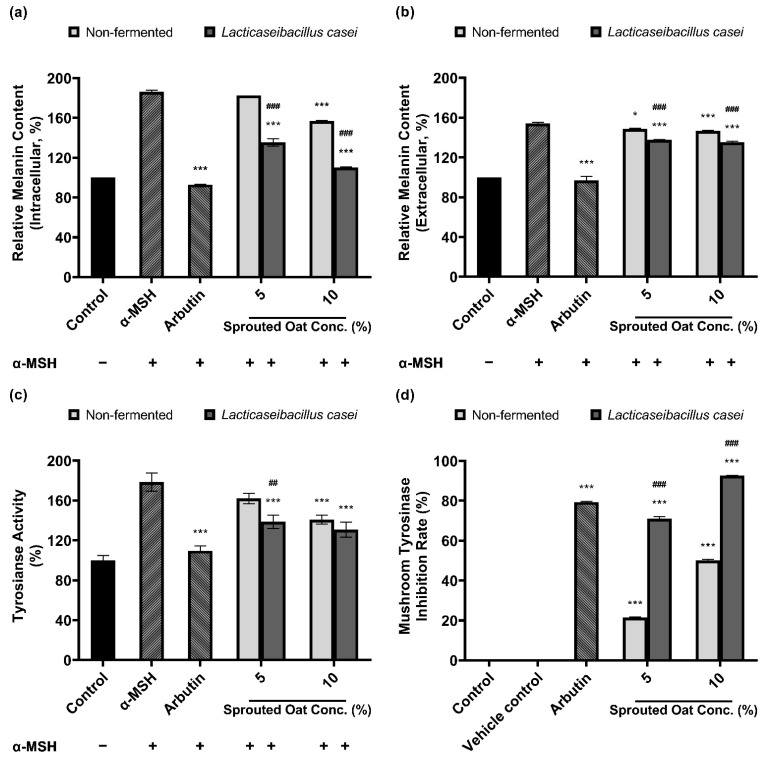
Inhibitory effect of non-fermented and *L. casei*-fermented sprouted oat extracts on melanin production in B16F10 cells. Relative (**a**) intracellular and (**b**) extracellular melanin contents. (**c**) Intracellular tyrosinase activity. α-MSH-stimulated B16F10 cells, excluding untreated control group, were treated with non-fermented and fermented sprouted oat extracts at a concentration of 1:100 for 48 h. Arbutin (500 µg/mL) was used as a positive control. Error bars show means ± SD. * *p* < 0.05, *** *p* < 0.001 compared to the α-MSH group. ## *p* < 0.01, ### *p* < 0.001 compared to the same concentration of non-fermented sprouted oat extracts. (**d**) Mushroom tyrosinase inhibition upon treatment with non-fermented and fermented sprouted oat extracts. Samples were treated at a concentration of 1:15. Arbutin (500 µg/mL) was used as a positive control, and tertiary distilled water filtered under the same conditions was used as a vehicle control. Error bars show means ± SD. *** *p* < 0.001 compared to the vehicle control. ### *p* < 0.001 compared to the same concentration of non-fermented sprouted oat extracts.

Figure 4a,b show that sprouted oat extracts reduced the amount of melanin in α-MSH-stimulated B16F10 cells. The amount of intracellular melanin increased to approximately 186% of the control value upon α-MSH stimulation and was considerably reduced by arbutin, which served as a positive control. Non-fermented sprouted oat extracts showed no significant reduction at a 5% concentration, whereas 5% *L. casei*-fermented sprouted oat extracts decreased the intracellular melanin content. At a concentration of 10%, the intracellular melanin content was reduced to 157.07% of the control value by the non-fermented sprouted oat extracts and to 110.33% by the *L. casei*-fermented sprouted oat extract. These results suggest that fermented sprouted oat extracts had a more significant attenuation effect than non-fermented sprouted oat extracts on α-MSH-stimulated intracellular melanin content at the same concentration (*p* < 0.001).

In addition to melanin synthesis, sprouted oat extracts reduced tyrosinase activity (Figure 4c). Intracellular tyrosinase activity in B16F10 cells increased upon α-MSH treatment and was reduced by the non-fermented sprouted oat extracts. Treatment with *L. casei*-fermented sprouted oat extracts showed a greater tyrosinase activity inhibition effect than treatment with non-fermented sprouted oats.

Furthermore, Figure 4d shows that *L. casei*-fermented sprouted oat extracts yielded a higher inhibition rate than the non-fermented sprouted oat extracts in the mushroom tyrosinase inhibition assay. Moreover, sprouted oat extracts inhibited mushroom tyrosinase. The non-fermented sprouted oat extracts showed inhibition rates of 21.39% and 50.11% at concentrations of 5% and 10%, respectively. *L. casei*-fermented sprouted oat extracts yielded 71.00% and 92.68% inhibition at 5% and 10% concentrations, respectively. Arbutin, used as a positive control, inhibited mushroom tyrosinase by 79.32%.

Collectively, the results showed that *L. casei*-fermented sprouted oat extracts provided significant benefits in terms of reducing intracellular and extracellular melanin content and intracellular tyrosinase activity, as well as inhibiting mushroom tyrosinase (*p* < 0.01). Moreover, the *L. casei*-fermented sprouted oat extracts induced an anti-melanogenesis effect.

### 3.5. Effects of L. casei-Fermented Oat Extracts on mRNA Expression of Genes Involved in Melanin Synthesis

**Figure 5:**
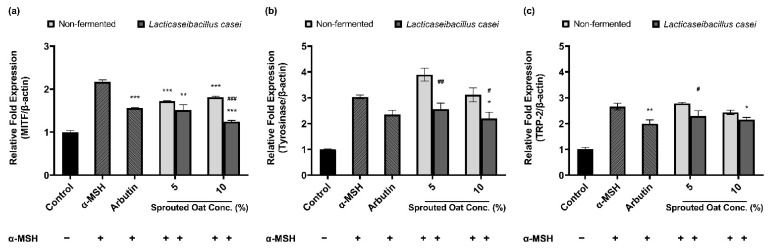
Effects of non-fermented and *L. casei*-fermented sprouted oat extracts on the relative mRNA expression of (**a**) MITF, (**b**) tyrosinase (TYR), and (**c**) TRP-2 in B16F10 cells. α-MSH-stimulated B16F10 cells, excluding the untreated control group, were treated with non-fermented and fermented sprouted oat extracts at a concentration of 1:100. Arbutin (500 µg/mL) was used as a positive control. Error bars show means ± SD. * *p* < 0.05, ** *p* < 0.01, *** *p* < 0.001 compared to the α-MSH group. # *p* < 0.05, ## *p* < 0.01, ### *p* < 0.001 compared to the same concentration of non-fermented sprouted oat extracts.

Figure 5 shows the effects of sprouted oat extracts on the mRNA expression of MITF, tyrosinase, and TRP-2, which are involved in melanin synthesis. α-MSH treatment increased the mRNA expression of MITF, tyrosinase, and TRP-2. *Lacticaseibacillus casei*-fermented sprouted oat extracts reduced the mRNA expression of MITF, tyrosinase, and TRP-2, but non-fermented sprouted oat extracts caused a reduction in expression only at the highest concentration (10%). In addition, *L. casei*-fermented sprouted oats suppressed MITF mRNA expression better than arbutin, which was used as a positive control.

### 3.6. Total Phenolic and Flavonoid Contents of Lactobacilli-Fermented Sprouted Oat Extracts

The total polyphenol and flavonoid contents of non-fermented and lactobacilli-fermented sprouted oat extracts at a concentration of 10% were measured (Table 2). Total phenolic contents were 3.05 ± 0.03 mg gallic acid equivalent (GAE) per 100 µL in non-fermented sprouted oat extracts and 4.75 ± 0.06 mg GAE/100 µL in *L. casei*-fermented sprouted oat extracts, with a significant difference between the two values (*p* < 0.001). Total flavonoid contents were 10.93 ± 0.14 mg quercetin equivalent (QE) per 100 µL in non-fermented sprouted oat extracts and 12.48 ± 0.05, 12.17 ± 0.16, 11.87 ± 0.05, and 12.60 ± 0.05 mg QE per 100 µL in *L. plantarum*-, *L. casei*-, *L. rhamnosus*-, and *L. gasseri*-fermented sprouted oat extracts, respectively, representing significant benefits compared with non-fermented sprouted oat extracts (*p* < 0.001). These results indicate that the total phenolic and flavonoid contents of the sprouted oat extracts increased through fermentation with lactobacilli.

The total phenolic and flavonoid contents of *L. casei*-fermented sprouted oat extracts at concentrations of 5% and 10% were shown to increase (Table 3). Total phenolic contents were 2.04 ± 0.05 and 2.83 ± 0.04 mg GAE/100 µL in 5% and 10% non-fermented sprouted oat extracts and 2.79 ± 0.12 and 4.57 ± 0.20 mg GAE/100 µL in 5% and 10% *L. casei*-fermented sprouted oat extracts, respectively, indicating a significant increase in the fermented extracts (*p* < 0.001). Total flavonoid content was 8.17 ± 0.18 and 10.45 ± 0.00 mg QE per 100 µL in 5% and 10% non-fermented sprouted oat extracts and 8.49 ± 0.05 and 11.45 ± 0.18 mg QE per 100 µL in 5% and 10% *L. casei*-fermented sprouted oat extracts. *Lacticaseibacillus casei*-fermented sprouted oat extracts showed no significant difference from non-fermented extracts in terms of phenolic and flavonoid contents at 5% but did show a significant increase in 10% extracts compared with the same concentration of non-fermented sprouted oat extracts (*p* < 0.001). These results suggest that the total phenolic and flavonoid contents of the sprouted oat extracts were increased by fermentation with *L. casei*.

### 3.7. Chemical Characteristics of Lactobacilli-Fermented Sprouted Oat Extracts

**Figure 6:**
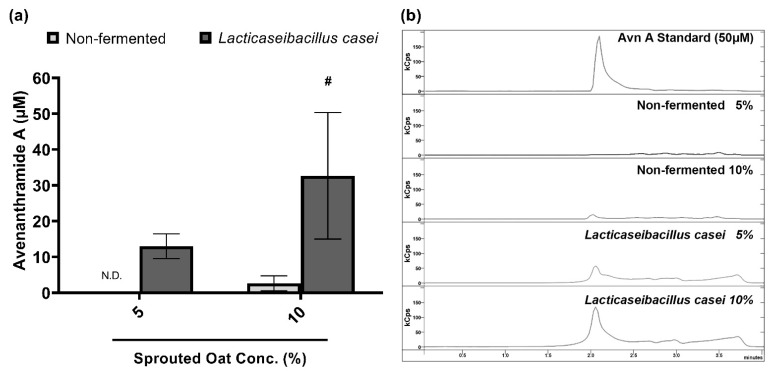
Avenanthramide A contents in non-fermented and *L. casei*-fermented sprouted oat extracts. (**a**) Quantification and (**b**) chromatogram of avenanthramide A in sprouted oat extracts. The abbreviation Avn A stands for avenanthramide A. N.D. means not detected. Error bars show means ± SD. # *p* < 0.05 compared to the same concentration of non-fermented sprouted oat extracts.

Avenanthramide A contents in sprouted oat extracts were quantified to confirm the component changes caused by fermentation. Figure 6 compares the contents of avenanthramide A in non-fermented and *L. casei*-fermented sprouted oat extracts. Avenanthramide A contents were significantly increased approximately 11-fold after the fermentation at a 10% concentration (*p* < 0.05). In detail, the total avenanthramide A contents were 2.67 µM in 10% non-fermented sprouted oat extracts and 32.67 µM in 10% *L. casei*-fermented sprouted oat extracts.

### 3.8. Antioxidant Activities of Lactobacilli-Fermented Sprouted Oat Extracts

**Figure 7:**
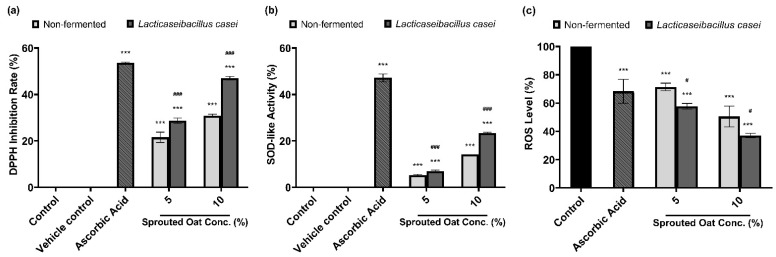
Effects of non-fermented and *L. casei*-fermented sprouted oat extracts on antioxidant activity. (**a**) DPPH radical-scavenging activity and (**b**) SOD-like activity. Ascorbic acid (50 µg/mL) was used as a positive control. Error bars show means ± SD. *** *p* < 0.001 compared with the vehicle control group. ### *p* < 0.001 compared with the same concentration of non-fermented sprouted oat extracts. (**c**) Cellular ROS levels, as measured by DCF-DA assay. B16F10 cells were treated with non-fermented and fermented sprouted oat extracts at a concentration of 1:100 for 24 h. Ascorbic acid (200 µg/mL) was used as a positive control. Error bars show means ± SD. *** *p* < 0.001 compared with the control. # *p* < 0.05 compared with the same concentration of non-fermented sprouted oat extracts.

DPPH radical-scavenging and SOD-like activities were measured to determine the antioxidant effects of the sprouted oat extracts. As shown in Figure 7a, 50 µg/mL ascorbic acid, used as a positive control, increased the DPPH radical-scavenging activity by 53.57% compared to the vehicle control group. The non-fermented sprouted oat extracts showed DPPH inhibition rates of 21.56% and 30.82% at 5% and 10% concentrations, respectively. *Lacticaseibacillus casei*-fermented sprouted oat extracts showed DPPH radical-scavenging activities of 28.70% and 47.02% at 5% and 10% concentrations, respectively, showing that the free radical-scavenging activity of *L. casei*-fermented sprouted oat extracts increased. In addition, the inhibition of autoxidation of pyrogallol was measured to determine the SOD-like activity. The fermented sprouted oat extracts suppressed the superoxide oxidation, exhibiting activity akin to SOD, an antioxidant enzyme (Figure 7b).

Furthermore, the protective effect of the fermented sprouted oat extracts on ROS levels in B16F10 cells within non-cytotoxic concentrations was monitored using the oxidation-sensitive dye DCF-DA. Figure 7c shows that fermented sprouted oat extracts more effectively suppressed ROS formation in B16F10 cells than non-fermented sprouted oat extracts (*p* < 0.05). This verified that *L. casei* fermentation increased the antioxidant effects of sprouted oat extracts.

## 4. Discussion

The health-promoting effects of natural ingredients have gained attention in various industries, including the pharmaceutical and cosmetic industries, as interest in health has increased owing to an increase in average life expectancy [57,58,59]. Research is ongoing on the antioxidant, anti-inflammatory, and anti-cancer effects of plant extracts. Moreover, the effects of plant extracts can be enhanced through fermentation techniques, which have long been used in the food and biotechnology fields [48]. This study aimed to investigate the antioxidant and anti-hyperpigmentation effects of sprouted oat extracts modified via fermentation. To achieve this, sprouted oat extracts were fermented using *L. plantarum*, *L. casei*, *L. rhamnosus*, and *L. gasseri*. *Lacticaseibacillus casei* fermentation, which yielded the most noticeable improvement in the tested parameters in sprouted oat extracts, was studied for its antioxidant and anti-melanogenic effects.

A fermentation process that involves the intentional conversion or modification of the substrate through microbial activity affects phenolic compounds, including polyphenols, flavonoids, vanillic acid, ferulic acid, and avenanthramides [60]. An increase in the total phenolic compounds in maca root extracts was reported through *Lactobacillus* spp. fermentation, and their antioxidant effect was increased [61]. Moreover, fermentation disrupts the cell wall matrix of plants and facilitates the extraction of phenolic compounds [60]. However, in a study on jujube puree, vanillic and ferulic acid contents decreased after fermentation [62]. This was assumed to occur due to the loss of heat-labile phenolic compounds and the degradation of polyphenols by lactic acid bacteria [62,63,64]. Even so, avenanthramide A, which showed the highest rate of content increase through germination among the three major isoforms of avenanthramides, is expected to withstand fermentation by being heat-stable and pH-stable [65,66,67]. Therefore, sprouted oats were fermented using lactobacilli to investigate the effects of fermentation on the total phenolic compounds, flavonoids, and avenanthramide A. In Table 2 and Table 3, the amount of the total phenolics and flavonoids in the fermented sprouted oat extracts was higher than in the non-fermented sprouted oat extracts. Fermented sprouted oat extracts showed different physiological activities depending on the strain, and fermentation with *L. casei* resulted in the greatest increase in the total phenolic compounds. Additionally, Figure 6 shows that fermented sprouted oat extracts contain more avenanthramide A compared to the non-fermented sprouted oat extracts at the same concentration. These findings emphasize that germination and fermentation are feasible processes for improving the health benefits of grains through the increased contents of phytochemicals. Although this study focused on changes in phenolic compounds and flavonoid content related to antioxidant effects, sprouted oats are composed of diverse elements. Therefore, it is expected that their widespread application will be possible if further investigation is conducted into the changes in the components of sprouted oats through fermentation.

Total phenolic compound levels are positively correlated with antioxidant activity [38,68,69]. To suppress oxidative stress, ROS-scavenging mechanisms, such as the inhibition of free radical formation and the chelation of metal ions, are required [70,71]. Phenolic compounds are known to inhibit the lipid peroxidation of cell membranes by inhibiting free radical reactions [72]. Furthermore, flavonoids inhibit the peroxidation of low-density lipoprotein and increase the absorption of vitamin C [72]. Vitamin C helps to reduce oxidized vitamin E, which in turn prevents the peroxidation of lipids in cell membranes [70,73,74]. Vitamin E, an antioxidant, is known to accumulate considerably in grains and leaves [70,74]. Moreover, the total phenolic compound and flavonoid contents in buckwheat sprouts increased following aging and fermentation, leading to a simultaneous enhancement in their antioxidant properties. This suggests that sprouted oat extracts with increased phenolic compound and flavonoid contents may have high antioxidant activity due to the fermentation [75]. Our findings show that *L. casei* fermentation increased the contents of antioxidant-related phytochemicals (Table 3) and enhanced DPPH radical-scavenging activity and SOD-like activity in acellular experiments (Figure 7a,b). Furthermore, the fermented sprouted oat extracts effectively reduced cellular ROS levels at non-cytotoxic concentrations, as shown in Figure 7c. Several studies have revealed that phytochemicals, including plant-derived polyphenols and avenanthramide, exert antioxidant effects through the Nrf2-dependent pathway [76,77,78]. Wine extracts abundant in polyphenols effectively reduced ROS levels induced by tertbutyl hydroperoxide in a dose-dependent manner. Further study indicated that this reduction was attributed to the activation of the Nrf2-ARE pathway, an essential antioxidant defense system, as evidenced by the increased nuclear Nrf2 in cells subjected to oxidative stress [79]. These studies suggested that the change in phytochemical content through fermentation is an important factor that can demonstrate antioxidant effects through the scavenging of ROS. These results might be due to the activation of the Nrf2-ARE pathway by *L. casei*-fermented sprouted oat extracts. Furthermore, the results of this study suggest that germination and lactobacilli fermentation are feasible processes for improving the health benefits based on the improved antioxidant capacity.

Antioxidants protect cells from oxidative stress and effectively inhibit aberrant melanogenesis [80,81,82,83]. UV rays cause photodamage to the skin, leading to melanin pigment production and oxidation, potentially causing hyperpigmentation. Therefore, a preventative effect against hyperpigmentation is expected if oxidation is suppressed or if a redox reaction is induced. *Lacticaseibacillus casei* is known to exhibit high antioxidant peptide production and to significantly improve antioxidant activity owing to its biosurfactant properties [84,85]. Therefore, we investigated whether sprouted oat extracts with enhanced antioxidant effects due to *L. casei* fermentation could inhibit melanogenesis. Melanogenesis is controlled by the rate-limiting enzyme tyrosinase [86]. Fermented sprouted oat extracts increased the tyrosinase inhibition rate and reduced tyrosinase activity, along with extracellular and intracellular melanin content, as shown in Figure 4. Moreover, tyrosinase and tyrosinase-related genes associated with anti-melanogenic activity are strictly controlled by MITF [86,87]. This suggests that a reduction in melanogenesis-related mRNA expression, especially that of MITF, has anti-hyperpigmentation effects [86]. Figure 5 shows that *L. casei*-fermented sprouted oat extracts reduced MITF, TYR, and TRP-2 mRNA expression. This is consistent with previous results showing that adlay and rice bran extracts fermented with *Lactobacillus* spp. downregulated MITF and reduced melanin production more effectively than non-fermented extracts [88,89]. Additionally, avenanthramides decreased tyrosinase activity in a dose-dependent manner and inhibited melanin production [90]. In addition, gallic acid, a phenolic compound that increased along with other phytochemicals during the fermentation of *Aronia melanocarpa*, decreased the protein expression levels of MITF, TRP-1, and TRP-2 in a dose-dependent manner. Moreover, the increased content of this phenolic compound was found to contribute to melanogenesis inhibition by upregulating p-AKT and p-GSK-3β and downregulating p-PKA and p-CREB in B16F10 cells [91]. These findings demonstrated that components of sprouted oats changed by *L. casei* fermentation may affect anti-melanogenesis. Accordingly, our results suggest that fermented sprouted oat extracts have the potential of these extracts to prevent hyperpigmentation and melanin overproduction. Further studies are needed to elucidate the cell signaling pathways involved in the inhibition of melanogenesis.

Overall, germination increases the nutritional value of grain products and promotes the biosynthesis of secondary metabolites. Additionally, the fermentation of sprouted grains improves various physiological activities through efficient bioconversion. These changes can lead to potential health benefits desired by the pharmaceutical and cosmetic industries. Furthermore, postbiotics produced through fermentation techniques are safer than probiotics and have antioxidant, antimicrobial, anti-inflammatory, and immunomodulatory effects [92,93]. Postbiotics are also attracting attention as raw materials that can be used in various industries due to their high stability and long shelf life [92]. Remarkably, research on the application of microbiome postbiotics to diet and cosmetics for skin improvement effects, such as anti-acne and wound healing, has been actively conducted [93,94]. These studies suggest that applying probiotics can lead to favorable potential health benefits desired by the pharmaceutical and cosmetics industries.

## 5. Conclusions

This study confirmed that the antioxidant and anti-melanogenic effects of sprouted oat extracts could be improved by fermentation with lactic acid bacteria. The phenolic compounds, flavonoids, and type A avenanthramide contents of the fermented sprouted oat extracts were significantly higher than those of the non-fermented sprouted oat extracts. In addition, increased DPPH radical-scavenging activity, SOD-like activity inhibition, and reduced ROS levels in cells suggested an improvement in the antioxidant effect through *Lactobacillus* fermentation. Furthermore, fermented sprouted oat extracts suppressed melanin overproduction by reducing MITF, tyrosinase, and TRP-2 mRNA expression and tyrosinase activity in B16F10 cells. These findings suggest that fermented sprouted oat extracts fermented by lactobacilli have the potential to be used as raw materials to protect skin from oxidative stress and melanin overaccumulation.

## Figures and Tables

**Table 1:** LC-MS/MS conditions.

Item	Conditions	
Column Column TeM	ACME C18 (50 mm × 2.1 mm, 1.9 µm) 30 °C	
Mobile Phase	A: 0.1% formic acid in water	B: 0.1% formic acid in methanol
Flow Rate Injection Volume	0.3 mL/min 2 µL	
Gradient	Time (min)	B (%)
	0	20
	2	90
	3	90
	3.1	20
	4	20
Ionization	HESI, positive mode	
Spray Voltage	5000 V	
Temperature	Cone: 350 °C	Heated Probe: 45 °C
Cone Gas Flow	20	
Heated Probe Gas Flow	0	
Nebulizer Gas Flow	8	

**Table 2:** Total Phenolic and Flavonoid Contents of Non-Fermented and Lactobacilli-Fermented Sprouted Oat Extracts.

Fermentation Treatment of 10% Sprouted Oat Extracts	Total Phenolic Content	Total Flavonoid Content
	(mg GAE/100 µL Extract)	(mg QE/100 µL Extract)
Non-fermented	3.05 ± 0.03	10.93 ± 0.14
*L. plantarum* KCTC 3108	3.17 ± 0.06	12.48 ± 0.05 ^###^
*L. casei* KCTC 3109	4.75 ± 0.06 ^###^	12.17 ± 0.16 ^###^
*L. rhamnosus* KCTC 5033	3.14 ± 0.09	11.87 ± 0.05 ^###^
*L. gasseri* KCTC 3143	3.15 ± 0.09	12.60 ± 0.05 ^###^

Data represent the means ± SD of three independent experiments. ^###^ *p* < 0.001 compared with the same concentration of non-fermented sprouted oat extract.

**Table 3:** Total Phenolic and Flavonoid Contents of Non-Fermented and *L. casei*-Fermented Sprouted Oat Extracts.

**Extract Concentration**	**Total Phenolic Content (mg GAE/100 µL Extract)**	
	**Non-Fermented**	***L. casei* KCTC 3109**
5%	2.04 ± 0.05	2.79 ± 0.12 ^###^
10%	2.83 ± 0.04	4.57 ± 0.20 ^###^
**Extract Concentration**	**Total Flavonoid Content (mg QE/100 µL extract)**	
	**Non-Fermented**	***L. casei* KCTC 3109**
5%	8.17 ± 0.18	8.49 ± 0.05
10%	10.45 ± 0.00	11.45 ± 0.18 ^###^

Data represent the means ± SD of three independent experiments. ^###^ *p* < 0.001 compared with the same concentration of non-fermented sprouted oat extracts.

## Data Availability

Data are contained within the article.
